# Progress in pH-Sensitive sensors: essential tools for organelle pH detection, spotlighting mitochondrion and diverse applications

**DOI:** 10.3389/fphar.2023.1339518

**Published:** 2024-01-10

**Authors:** Shu-Ang Li, Xiao-Yan Meng, Ying-Jie Zhang, Cai-Li Chen, Yu-Xue Jiao, Yong-Qing Zhu, Pei-Pei Liu, Wei Sun

**Affiliations:** ^1^ Clinical Systems Biology Laboratories, The First Affiliated Hospital of Zhengzhou University, Zhengzhou, China; ^2^ The Academy of Medical Sciences, Zhengzhou University, Zhengzhou, China; ^3^ Department of Immunology, School of Basic Medical Sciences, Xinxiang Medical University, Xinxiang, Henan, China; ^4^ Department of Burn and Repair Reconstruction, The First Affiliated Hospital of Zhengzhou University, Zhengzhou, China

**Keywords:** pH-sensitive, sensors, mitochondrion, organelle pH, disease progression

## Abstract

pH-sensitive fluorescent proteins have revolutionized the field of cellular imaging and physiology, offering insight into the dynamic pH changes that underlie fundamental cellular processes. This comprehensive review explores the diverse applications and recent advances in the use of pH-sensitive fluorescent proteins. These remarkable tools enable researchers to visualize and monitor pH variations within subcellular compartments, especially mitochondria, shedding light on organelle-specific pH regulation. They play pivotal roles in visualizing exocytosis and endocytosis events in synaptic transmission, monitoring cell death and apoptosis, and understanding drug effects and disease progression. Recent advancements have led to improved photostability, pH specificity, and subcellular targeting, enhancing their utility. Techniques for multiplexed imaging, three-dimensional visualization, and super-resolution microscopy are expanding the horizon of pH-sensitive protein applications. The future holds promise for their integration into optogenetics and drug discovery. With their ever-evolving capabilities, pH-sensitive fluorescent proteins remain indispensable tools for unravelling cellular dynamics and driving breakthroughs in biological research. This review serves as a comprehensive resource for researchers seeking to harness the potential of pH-sensitive fluorescent proteins.

## Introduction

Cellular processes are intricately regulated by pH dynamics, with precise pH levels being critical for various biological functions. Monitoring pH changes within living cells is essential for understanding the underlying mechanisms of physiological processes, disease progression, and drug effects. pH-sensitive fluorescent proteins have emerged as versatile and indispensable tools for visualizing and quantifying pH fluctuations in real time via noninvasive techniques.

In this review, we delve into the diverse and dynamic world of pH-sensitive fluorescent proteins, highlighting their pivotal role in cellular imaging and physiology. These proteins ushered in a new era of research, enabling scientists to gain insights into the spatial and temporal variations in pH within subcellular compartments. By offering a unique way to track pH dynamics in a living system, pH-sensitive fluorescent proteins have paved the way for an array of applications spanning various fields of biology and medicine. In our review we list the different types of pH-sensitive fluorescent proteins available, including the pioneering green fluorescent protein (GFP)-based variants, such as GFP S65T, ratiometric pHluorin, superecliptic pHluorin, T-sapphire, deGFP4, E^1^GFP, E^2^GFP, pHluorin2, DendFP and Dendra2; red fluorescent protein (RFP)-based variants, such as mOrange2, mApple, mNectarine, pHRed, pHTomato, pHoran4, pHuji, and pHScarlet; yellow fluorescent protein and the YFP-based variants, SypHer, SypHer2, SypHerRed, and mNeonGreen; and cyan fluorescent protein (CFP)-based variants, such as mTFP1 and ECFP. In addition, other pH sensors, including pHlameleons, pHCECnSensor01, pH-Lemon, Tandem, pHLARE, and EGF-CoGFP-mTagBFP2/mCRISPRed, were used in the field. Each of these proteins possesses distinct spectral properties, pH-sensing ranges, and pH-dependent fluorescence responses, and their excitation wavelengths (λex), emission wavelengths (λem), extinction coefficients (Ec), fluorescence quantum yields (QYs), brightness, and pKa values are summarized in Table 1.

**TABLE 1:** The spectral characteristics of pH-sensitive fluorescent proteins.

	FP	λex	λem	Ec	QY	Brightness	pKa	References
GFP	GFP S65T	490	510	55,000	0.64	35.2	-	Heim et al. (1995)
	Ratiometric pHluorin	475/395	509	-	-	-	7.1	Miesenböck et al. (1998)
	Superecliptic pHluorin	495	512	-	-	-	7.2	Shen et al. (2014)
	pHGFP	410/470	535	-	-	-	-	Moseyko and Feldman (2001)
	T-sapphire	399	511	44,000	0.6	26.4	4.9	Zapata-Hommer and Griesbeck (2003)
	deGFP4	509	518	26,900	0.15	4.04	7.37	Hanson et al. (2002)
	Pt-GFP	390/502	508	-	-	-	7.3	Schulte et al. (2006)
	E^1^GFP	488	507	55,900	0.6	33.54	6.0	Serresi et al. (2009)
	E^2^GFP (Acid)	424	510	31,500	0.22	-	-	Bizzarri et al. (2006)
	E^2^GFP (Alkaline)	401/515	523	29,280	0.91	-	-	Bizzarri et al. (2006)
	pHluorin2	475/395	509	-	-	-	-	Mahon (2011), Valkonen et al. (2013)
	PepHlurion/PrpHlurion	395/475	512/515	-	-	-	-/6.6	Shen et al. (2013)
	CoGFP	388/498	456/507	-	-	-	6.5	Ogoh et al. (2013)
	DendGFP	492	508	90,000	0.65	58.5	6.5	Pakhomov et al. (2015)
	DendRFP	557	575	35,000	0.68	23.8	5.2	Pakhomov et al. (2015)
	Dendra2 (Green)	490	507	45,000	0.5	22.5	6.6	Pakhomov et al. (2017)
	Dendra2 (Red)	553	573	35,000	0.55	19.25	6.9	Pakhomov et al. (2017)
RFP	mOrange2	549	565	58,000	0.60	35	6.5	Shaner et al. (2008)
	mApple	568	592	75,000	0.49	37	6.5	Shaner et al. (2008)
	mNectarine	558	578	58,000	0.45	26	6.9	Johnson et al. (2009)
	mKate	588	635	45,000	0.33	67	6.2	Shcherbo et al. (2007)
	mKate2	588	635	62,500	0.40	74	5.4	Shcherbo et al. (2009)
	pHRed	440/585	620	-	-	-	6.9	Tantama et al. (2011)
	pHTomato	550	580	-	-	-	7.8	Li and Tsien (2012)
	pHoran4	547	561	-	-	-	7.5	Shen et al. (2014)
	pHuji	572	598	31,000	0.22	6.82	7.7	Shen et al. (2014), Liu et al. (2021a)
	pHmScarlet	562	585	85,000	0.47	39.73	7.4	Liu et al. (2021a)
YFP	YFP	392/514	528	-	-	-	7.0	Elsliger et al. (1999)
	EYFP	390/514	527	-	-	-	7.1	Llopis et al. (1998)
	mCitrine	516	529	77,000	0.76	-	5.7	Griesbeck et al. (2001)
	mtAlpHi	498	522	-	-		8.5	Abad et al. (2004)
	SypHer	420/490	535	-	-	-	-	Poburko et al. (2011)
	SypHer2	427/504	525	-	-	-	-	Matlashov et al. (2015)
	SypHerRed	575	605	-	-	-	-	Shimolina et al. (2022)
	mNeonGreen	506	517	116,000	0.8	92.8	10.0	Tutol et al. (2019)
CFP	mTFP1	462	492	64,000	0.85	54	4.3	Ai et al. (2006), Ai et al. (2008)
	ECFP	435	474	30,000	0.30	9.0	4.7	Lelimousin et al. (2009)

One of the notable applications discussed in this review is their role in studying organelle pH, elucidating the pH values associated with different cellular compartments. Notably, aberrant pH variations within cellular organelles, such as mitochondria, have been closely correlated with the onset of serious ailments such as cancer (Vyas et al., 2016) and neurodegenerative diseases (Devine and Kittler, 2018). Similarly, deviations in the pH of the Golgi apparatus can lead to abnormal glycosylation, which has been associated with the development of cancers and cutis laxa formation (Rivinoja et al., 2012). Dysregulation of the pH within lysosomes may result in lysosomal dysfunction, potentially contributing to various diseases, including neurodegenerative diseases (Wang et al., 2018), inflammation, autoimmune diseases (Ge et al., 2015), and disorders of lipid and glucose metabolism (Gu et al., 2021), among others. The ability to monitor the pH of the cytosol, nucleus, mitochondria, endoplasmic reticulum, Golgi apparatus, lysosomes, and peroxisomes has deepened our understanding of organelle-specific pH regulation, as well as their applications in studying intracellular pH regulation, vesicle trafficking, and membrane fusion events.

Furthermore, these proteins have been instrumental in visualizing exocytosis and endocytosis processes, particularly in the context of synaptic transmission. pH-sensitive fluorescent proteins, such as superecliptic pHluorin and synapto-pHluorin, have allowed researchers to precisely monitor transmission events at individual synaptic boutons. The ability of these vesicles to detect both exocytosis and endocytosis has provided invaluable insights into synaptic communication. In addition, these pH sensors have found diverse applications in understanding the effects of drugs and the progression of diseases, detecting cell death and apoptotic processes, and monitoring nucleoside transport.

As an ever-evolving field, this review also covers recent advances and future directions in the use of pH-sensitive fluorescent proteins. Improved photostability, pH specificity, and subcellular targeting capabilities have enhanced their utility. Novel techniques, including multiplexed imaging, three-dimensional visualization, and super-resolution microscopy, are expanding their applications. Additionally, the review sheds light on potential applications in optogenetics and drug discovery.

This review provides a comprehensive reference for researchers interested in harnessing the potential of pH-sensitive fluorescent proteins. With their unparalleled ability to uncover the intricacies of cellular physiology and disease mechanisms, pH-sensitive fluorescent proteins continue to drive progress in biological research, providing insight into the dynamic nature of pH within living cells. pH-sensitive fluorescent proteins have revolutionized the field of cellular imaging by providing a unique window into the dynamic nature of intracellular pH changes. These remarkable biomolecular tools have enabled researchers to visualize and monitor pH fluctuations in real time, shedding light on various biological processes.

In this comprehensive review, we explore the diverse types, mechanisms, applications, and recent advancements of pH-sensitive fluorescent proteins, highlighting their significant contributions to our understanding of cellular dynamics.

## Types of pH-sensitive fluorescent proteins

### Green fluorescent protein variants

***GFP S65T.*** GFP S65T is a mutation introduced in the jellyfish green fluorescent protein fluorophore (S65-Y66-G67). The S65T mutation significantly improved the pH sensitivity of GFP across a pH range of 5.0–8.0 (Elsliger et al., 1999). Additionally, it enhances the GFP fluorescence intensity and photostability while shifting the major excitation peak from 395 nm to near 490 nm (Heim et al., 1995; Heim and Tsien, 1996). These changes effectively address the shortcomings of the wild-type protein, increasing its suitability for research applications.

***Ratiometric pHluorin.*** Ratiometric pHluorin, a pH-sensitive fluorescent protein in the GFP family, exhibits pH-dependent fluorescence. Derived from GFP with the S202H mutation, this protein exhibited a 16% decrease in intensity at 395 nm excitation and a 26% increase at 475 nm upon a pH shift from 7.4 to 6.0. Additional mutations (E132D, S147E, N149L, N164I, K166Q, I167V, R168H, and L220F) enhance its properties. Unlike conventional pHluorin, it uses dual wavelengths (395 nm and 475 nm) for ratiometric measurements, providing accurate pH assessments in cellular compartments (Miesenböck et al., 1998). The pHluorin M153R mutation maintains pH-dependent behavior, enhancing fusion protein stability without altering excitation ratios, making it a bright and reliable tool for live cell pH measurements and protein distribution analysis (Morimoto et al., 2011).

***Superecliptic pHluorin.*** Superecliptic pHluorin (SEP), derived from pHluorin, enhances fluorescence to visualize cellular processes, notably membrane fusion and neurotransmitter release. Its name reflects improved pH sensitivity and brightness, enabling precise monitoring of pH changes in subcellular compartments or at the cell surface. Unlike ratiometric pHluorin, SEP lacks the S202H mutation but features substitutions such as Q80R, S147D, N149Q, T161I, S202F, Q204T, and A206T (Miesenböck et al., 1998). Used in live-cell imaging, SEP tracks vesicle trafficking, exocytosis, and endocytosis, offering insights into cellular communication. The SEP A227D mutant retains pH responsiveness and enhances the voltage-dependent signal of Genetically Encoded Voltage Indicators (GEVI) (Kang et al., 2019).

***pHGFP.*** pHGFP was first optimized from the ratiometric pHluorin, which can be expressed in *Arabidopsis thaliana* and tobacco plants. Using this pH-sensitive protein was suitable for monitoring the pH of cytosolic dynamics in individual plant cells as well as in whole plant tissues. The results revealed significant differences in the pH gradient between different developmental regions of the roots of *Arabidopsis thaliana* (Moseyko and Feldman, 2001).

***T-Sapphire.*** T-Sapphire, an intrinsically fluorescent protein originating from the sapphire variant of GFP, was distinguished by the lack of a second excitation peak at 475 nm in the wild-type GFP due to the T203I mutation. T-Sapphire incorporates the mutations Q69M, C70V, V163A, and S175G. Notably, both T-Sapphire and Sapphire exhibited significant stokes shifts, characterized by an excitation peak at 399 nm and an emission peak at 511 nm. T-Sapphire exhibits sensitivity to pH variations, maintaining its absorbance stability within the pH range of 4.0–7.0. A distinctive feature of its fluorescence is its pKa value of 4.9, suggesting high resilience to pH fluctuations within the typical pH 6.8 to 7.3 cytosolic environment of live cells (Zapata-Hommer and Griesbeck, 2003). This intrinsic attribute facilitates the visualization of slightly acidic compartments, such as the Golgi or secretory vesicles using this protein.

***deGFP4.*** deGFP4 is a recently engineered variant of the green fluorescent protein encompassing the S65T/C48S/H148C/T203C site. This structural arrangement bestows upon it the unique traits of dual emission capability and sensitivity to pH variations with a pKa value of approximately 7.3. Through confocal microscopy, the alterations in the emission ratio of deGFP4 were closely observed, demonstrating a dynamic range akin to that of the commercially accessible pH-sensitive dye SNARF-1 in PS120 cells. Moreover, deGFP4 showed notable superiority in terms of fluorescent signal intensity over cellular autofluorescence when subjected to two-photon excitation, surpassing the capabilities of traditional confocal microscopy. With its favorable optical characteristics, appropriate pKa values spanning the physiological pH spectrum, and compatibility with ratiometric assessments, the dual-emission functionality of deGFP4 presents an immensely promising avenue for serving as a probe for pH investigations *in vivo* (Hanson et al., 2002).

***Pt-GFP.*** Pt-GFP is a newly developed fluorescent pH reporter derived from the orange seapen *Ptilosarcus gurneyi*. It offers a wider pH responsiveness range, an excellent dynamic ratio range, and enhanced acid stability. When expressed in *Arabidopsis thaliana*, Pt-GFP can effectively regulate both cytosolic pH regulation and changes in response to conditions such as anoxia and salt stress (Schulte et al., 2006).

***E* ^*1*^ *GFP.*** E^1^GFP, commonly referred to as enhanced green fluorescent protein, represents a modified rendition of the original green fluorescent protein. This modified version is distinguished by the incorporation of the F64L and T203Y mutations, which are strategically employed to increase the protein’s folding efficiency at a physiological temperature of 37°C. The term “ratiometric by emission” describes E^1^GFP’s inherent ability to undergo changes in its emission characteristics directly in response to pH shifts. Essentially, the fluorescence emitted by E^1^GFP can be gauged at two distinct wavelengths, with the ratio of intensities at these wavelengths contingent upon the prevailing pH within the protein environment. With a pKa close to 6.0, E^1^GFP is particularly well suited for detecting pH levels that lean toward the acidic end of the spectrum (Serresi et al., 2009).

***E* ^*2*^ *GFP.*** The F64L/S65T/T203Y/L231H GFP mutant, termed E^2^GFP, has been developed as a ratiometric pH indicator for studying intracellular conditions. E^2^GFP has two spectral forms that change with pH under both excitation and emission conditions, with a pKa value of approximately 7.0. Optimal excitation occurs at 488 and 458 nm, providing a strong signal dynamic range and ratiometric deviation from the thermodynamic pK. This makes E^2^GFP suitable for imaging setups with common light sources and filter configurations. E^2^GFP was utilized to measure the average intracellular pH and spatial pH in CHO and U-2 OS cell lines under normal circumstances. Additionally, it was used to monitor pH changes during mitosis in CHO cells and to target specific subcellular regions, such as nucleoli and nuclear promyelocytic leukemia bodies (Bizzarri et al., 2006).

***pHluorin2.*** pHluorin2 represents a significant improvement over the original ratiometric pHluorin, as it is constructed from GFP2 with the incorporation of mammalian codons, the F64L mutation, and key pHluorin-specific mutations. pHluorin2 retains ratiometric pH sensitivity while significantly boosting fluorescence. Unlike native pHluorin, pHluorin2 within the ligand-binding domain of the parathyroid hormone 1 receptor is easily detectable through confocal microscopy and displays heightened fluorescence during ligand-triggered endocytosis. pHluorin2 offers enhanced fluorescence and maintained ratiometric pH sensitivity, marking a substantial advancement in this research approach (Mahon, 2011).

***PEpHluorin and PRpHluorin.*** PEpHluorin and PRpHluorin play crucial roles as pH sensors in plant cells, enabling the measurement of pH in different intracellular compartments. When these sensors were used, the study revealed that the cytosol and nucleus exhibited similar pH values of approximately 7.3 and 7.2, respectively, while other organelles, such as peroxisomes, the mitochondrial matrix, and the plastidial stroma, maintained an alkaline pH. Along the secretory pathway, progressive acidification was observed, ranging from pH 7.1 in the endoplasmic reticulum to pH 5.2 in the vacuole, with the trans-Golgi network (TGN) and multivesicular body (MVB) exhibiting similar pH values of approximately 6.3 and 6.2, respectively. Interestingly, inhibiting vacuolar H^+^-ATPase (V-ATPase) with concanamycin A induced a significant increase in pH within the TGN and vacuole. These two pH sensors are invaluable tools for visualizing and precisely quantifying pH variations within plant cells (Shen et al., 2013).

***CoGFP.*** CoGFP, a green fluorescent protein (GFP) derived from the sea cactus *Cavernularia obesa*, naturally exists as a dimer, with well-defined absorption peaks at 388 nm and 498 nm. When excited at 388 nm, it emits blue fluorescence at pH values less than 5 and green fluorescence at pH values greater than 7, demonstrating stability at pH 4. When subjected to 498 nm excitation, the probe emits green fluorescence within the pH range of 5–9. Researchers have ingeniously transformed CoGFP into a monomeric form, departing from its original dimeric structure. This monomeric version has proven invaluable for tracking intracellular pH variations during the phagocytosis of living cells using fluorescence microscopy. This pioneering approach streamlines the process through single-wavelength excitation and a longpass emission filter, making it more technically accessible than dual-wavelength excitation with dual-emission fluorescent proteins (Ogoh et al., 2013).

***DendFP.*** DendFP was introduced as a tetrameric green fluorescent protein obtained from *Dendronephthya* sp. in 2002*.* This photoconvertible protein is a Kaede-like fluorescent protein that displays distinct characteristics (Labas et al., 2002). Unlike many existing genetically encoded pH sensors, DendFP does not experience fluorescence quenching with increased acidity. Instead, its emission peak transitions from red to green. Consequently, pH can be quantitatively assessed by analysing the emission intensity ratio between the red and green spectra. This unique characteristic sets DendFP apart from other pH sensors (Pakhomov et al., 2015).

***Dendra2.*** Dendra2, a monomeric GFP-like protein and a variant of the photoconvertible fluorescent protein DendFP, is classified within the Kaede-like group of photoconvertible fluorescent proteins (Labas et al., 2002). It undergoes an irreversible transformation from green to red upon exposure to violet-blue light. The red-emitting state transitions to green in acidic environments due to the protonation of the chromophore’s phenolic group. This inherent reversibility makes Dendra2 a promising candidate for ratiometric pH sensing within the physiological pH range, as it features a pKa of 7.1 and 7.5 before and after photoreaction, respectively (Pakhomov et al., 2017).

### Red fluorescent protein variants

***mOrange2.*** mOrange2, an engineered variant of the mOrange fluorescent protein, was developed through the introduction of specific mutations (Q64H, F99Y, E160K, and G196D) (Shaner et al., 2008). In contrast to mOrange, mOrange2 exhibits a remarkable 6-fold increase in photostability, rendering it well-suited for fusion applications within mammalian cells. However, compared with its predecessor, mOrange2 displayed a slight 30% reduction in brightness. Notably, its excitation and emission peaks experienced subtle shifts to 549 nm and 565 nm, respectively. Despite these alterations, mOrange2 maintains its responsiveness to acidity, characterized by a pKa value of 6.5. This feature positions mOrange2 as a valuable candidate for labelling acidic compartments, thereby enhancing its potential as a marker for processes involving exocytosis or other pH-dependent phenomena (Sarker et al., 2022).

***mApple.*** mApple, an engineered protein resulting from the fusion of DsRed originates from a coral anemone (*Discosoma* sp.) (Matz et al., 1999; Shaner et al., 2008). Notably, mApple is pH responsive and functions effectively across the pH range from 4.6 to 7.4. This characteristic enables accurate and precise quantification of subcellular pH. The pH-sensitive protein mApple has been designed to specifically target the cytosol, endosomes, and lysosomes. This strategic localization renders mApple a central component of rapid pH-dependent fluorescent lifetime imaging microscopy (pHLIM), facilitating accurate evaluation of subcellular pH levels (Rennick et al., 2022).

***mNectarine.*** mNectarine, a monomeric red fluorescent protein, originates from *Discosoma* sp. and is a pH-sensitive mFruit variant (Johnson et al., 2009). It was developed through iterative rounds of directed evolution using random mutagenesis. With a pKa of 6.98, mNectarine is well-suited for tracking physiological pH changes in mammalian cells. By fusing mNectarine with human concentrative nucleoside transport 3 (hCNT3) at its N-terminus, the pH was measured at the intracellular surface of hCNT3. This fusion approach represents an effective technique for monitoring nucleoside transport, suggesting its potential application for assessing the activity of other transport proteins reliant on H^+^ coupling (Johnson et al., 2009).

***mKate.*** mKate, a monomeric derivative originating from Katushka, is celebrated for its exceptional brightness and photostability, rendering it a premier choice for fluorescent protein labelling within the far-red spectrum. Furthermore, mKate shows rapid and complete chromophore maturation at 37°C, with a half-life as short as 75 min. Its fluorescence remains remarkably stable at pH 6.0 and below, featuring a pKa value of 6.0 (Shcherbo et al., 2007).

***mKate2.*** mKate2, a monomeric far-red fluorescent protein, surpasses its predecessor, mKate, in terms of brightness, facilitating enhanced multicolor labelling and whole-body imaging. This novel protein shows high brightness, a far-red emission spectrum, pH resistance, and photostability, making it an ideal choice for live tissue imaging in cells and animals (Shcherbo et al., 2009). This advancement broadens the possibilities for research in the field of fluorescent proteins.

***pHRed.*** pHRed is a red fluorescent protein (RFP) engineered from the long Stokes shift FP mKeima, and incorporates the A213S mutant for pH sensitivity (Violot et al., 2009). Notably, this approach has emerged as a pioneering single-protein, ratiometric red fluorescent sensor developed for pH detection. Its fluorescence emission peak at 610 nm was coupled with dual excitation peaks at 440 nm and 585 nm, facilitating ratiometric imaging. The intensity ratio varies with an apparent pKa of 6.6, exhibiting a dynamic range exceeding 10-fold. Additionally, pHRed presented a pH-responsive fluorescence lifetime shift of approximately 0.4 ns across physiological pH values, which was detectable via single-wavelength two-photon excitation. Furthermore, pHRed has proven successful in monitoring intracellular pH fluctuations through imaging energy-dependent shifts in both cytosolic and mitochondrial pH (Tantama et al., 2011).

***pHTomato.*** pHTomato, derived from the monomeric variant mRFP/mStrawberry (Campbell et al., 2002; Shaner et al., 2004), shares similar excitation (550 nm) and emission (580 nm) peaks with tomato variants of the mFruit series (Shaner et al., 2004). It differs from mStrawberry through six amino acid substitutions (F41T, F83L, S182K, I194K, V195T, and G196D). Notably, pHTomato features a crucial threonine at position 66, reminiscent of mRFP-Q66T (Jach et al., 2006). A significant attribute is its heightened sensitivity to pH, with a pKa value of approximately 7.8. Furthermore, the fusion of pHTomato and GFP-based probes with different variants of the channel rhodopsin introduced an all-optical method for multiplex control and tracking of distinct circuit pathways (Li and Tsien, 2012).

***pHoran4.*** pHoran4 (pH-sensitive orange FPs) is an offshoot of the pH-sensitive mOrange protein and is distinguished by the M163K mutation in close proximity to the chromophore. This mutation clearly demonstrated the crucial role of the residue in modulating the pH sensitivity of *Discosoma* red fluorescent protein variants. Its pKa, measuring 7.5, closely mirrors that of superecliptic pHluorin. However, it is important to note that the change in fluorescence intensity from pH 5.5 to 7.5 remains notably smaller than that of superecliptic pHluorin (Shen et al., 2014).

***pHuji.*** pHuji is an enhanced variant derived from the red fluorescent protein mApple K163Y (pronounced “Fuji”, akin to an apple cultivar). This variant exhibited a remarkable more than 20-fold alteration in fluorescence intensity between pH 5.5 and 7.5. Moreover, the pHuji emission peak at 598 nm exhibited negligible pH-dependent shifting and was distinctly separated from the superecliptic pHluorin emission at 512 nm. These attributes collectively facilitate simultaneous two-color imaging in conjunction with the superecliptic pHluorin, thus making pHuji suitable for detecting single exocytosis and endocytosis events (Shen et al., 2014).

***pHScarlet.*** pHScarlet is an enhanced version of the mScarlet fluorescent protein that was meticulously designed to exhibit pH sensitivity. Upon emission of vibrant red fluorescence, an excitation peak at approximately 568 nm and an emission peak near 592 nm were observed. This heightened pH responsiveness enables the concurrent monitoring of vesicle docking and fusion events. pHmScarlet can also seamlessly pair with superecliptic pHluorin, facilitating dual-color imaging of two discrete secretory events: vesicle exocytosis and docking. Notably, pHmScarlet has the ability to maintain exceptional spatial resolution, despite its emission wavelength being redshifted when compared to that of superecliptic pHluorin. This attribute holds particular significance when utilizing Hessian-structured illumination microscopy (Hessian-SIM) to uncover the intricate ring structure of vesicle fusion pores (Liu A. et al., 2021).

### Yellow fluorescent protein variants

***YFP.*** Like that of GFP S65T, the pH dependence of yellow fluorescent protein (GFP S65G/V68L/S72A/T203Y) was also notable. Specifically, YFP H148G introduces changes in solvent accessibility (Wachter et al., 1998), while YFP H148Q and YFP E222Q modify polar groups near the chromophore. pH titration assays performed on these YFP variants revealed the ability to adjust the chromophore’s pKa across a wide spectrum, ranging from 6.0 to 8.0. Consequently, this range expansion allows accurate pH determination at pH values ranging from 5.0 to 9.0 (Elsliger et al., 1999).

***EYFP.*** EYFP (enhanced yellow fluorescent protein), a variant of GFP, has pH-dependent absorbance and fluorescence. By targeting EYFP to specific cell compartments, it becomes possible to measure the pH in the cytosol, nucleus, Golgi (ET-EYFP), and mitochondrial matrix (EYFP-mito). These observations indicate that the Golgi membrane is permeable to hydrogen ions (H^+^) and that chloride ions (Cl^−^) appear to contribute to maintaining electroneutrality during H^+^ transport (Llopis et al., 1998).

***mCitrine.*** mCitrine, a novel yellow variant of green fluorescent protein, has been engineered in *Escherichia coli*. Compared with its predecessors, mCitrine offers numerous advantages, including reduced sensitivity to pH changes, resistance to chloride interference, improved photostability, and enhanced expression within organelles at 37°C. The heightened resistance to halides is attributed to structural modifications. Additionally, mCitrine has been pivotal in the development of improved calcium indicators that can be selectively targeted to various cellular locations. This breakthrough enables the very first single-cell visualization of free calcium concentrations in the Golgi. mCitrine has been shown to be highly effective across a wide range of applications, particularly in the field of genetically encoded fluorescent indicators for monitoring physiological signals (Griesbeck et al., 2001).

***mtAlpHi.*** mtAlpHi is a green fluorescent protein chimera that is specifically designed for high sensitivity to alkaline pH levels. This innovative probe serves as a mitochondrial alkaline pH indicator and is expertly tailored to investigate mitochondrial matrix pH dynamics. mtAlpHi displays remarkable sensitivity, with an apparent pKa of approximately 8.5, and exhibits reversible and substantial changes in fluorescence in response to pH shifts, both in controlled laboratory settings and within living cells. It is purposefully directed to the mitochondrial matrix, facilitating the tracking of pH fluctuations in diverse scenarios, including the use of uncouplers, Ca^2+^ ionophores, medications affecting ATP synthesis or electron flow, weak acids or bases, and receptor activation (Abad et al., 2004).

***SypHer.*** SypHer (a synthetic pH sensor) is a pH-sensitive yellow fluorescent protein that contains the C199S mutation of HyPer (Poburko et al., 2011). This design incorporates circularly permuted yellow fluorescent protein (cpYFP) inserted into the regulatory domain of the prokaryotic H_2_O_2_-sensing protein OxyR (Belousov et al., 2006).

***SypHer2.*** SypHer-2, a variant of HyPer-2, introduces the C199S substitution. HyPer-2 itself is a HyPer mutant featuring the single point mutation A406V, equivalent to A233V in the wild-type OxyR (Markvicheva et al., 2011). Notably, compared with those of SypHer, the fluorescence signals of the NIH/3T3 and HeLa-Kyoto cell lines were significantly brighter. Although the pH sensitivities of SypHer and SypHer-2 appear identical, SypHer2 proves useful for monitoring pH at both presynaptic and postsynaptic termini, revealing substantial dynamic differences in pH between synaptic boutons and dendritic spines (Matlashov et al., 2015).

***SypHerRed.*** SypHerRed originates from the circularly permuted red fluorescent protein mApple and is engineered through the introduction of the C199S mutation into the hydrogen peroxide biosensor HyPerRed (Ermakova et al., 2014). Notably, SypHerRed exhibited a redshifted spectrum with an excitation maximum at 575 nm and an emission maximum at 605 nm. These characteristics render it suitable for visualizing the intracellular pH (pHi) in cancer cell cultures and mouse tumor xenografts. This visualization can be achieved through techniques such as fluorescence lifetime imaging microscopy and macroscopy (FLIM) (Shimolina et al., 2022).

***mNeonGreen.*** mNeonGreen, the most luminous monomeric green/yellow fluorescent protein, is derived from the tetrameric yellow fluorescent protein lanYFP found in the *cephalochordate B. lanceolatum*. It exhibits pH sensitivity within the range of pH 4.0 to pH 8.0 and serves as an effective turn-on fluorescent protein sensor for chloride (Tutol et al., 2019). When conducting fluorescence measurements, the mNeonGreen fluorophore can be excited using a 480 nm laser and its emission can be detected at 517 nm. This particular fluorescent protein stands out as the most luminous monomeric green or yellow fluorescent protein currently known. It excels as a fusion tag in both conventional imaging techniques and stochastic single-molecule super resolution imaging. Moreover, it serves as an outstanding FRET acceptor, particularly when paired with the latest generation of cyan fluorescent proteins (Shaner et al., 2013).

### Cyan fluorescent protein variants

***mTFP1.*** mTFP1 (monomeric teal FP1) originates from the tandem dimer (td) group of fluorescent proteins and was initially derived from the tetrameric cyan fluorescent protein cFP484 found in *Clavularia coral*. This particular protein exhibited singular and vibrant cyan fluorescence when in a monomeric state, which was achieved through cyan excitation at 462 nm and subsequent emission at 492 nm. Despite having a modest pKa value of 4.3, mTFP1 displays sensitivity to pH fluctuations within the range of 2.0–7.0; nevertheless, its fluorescence remains unaltered amidst physiologically relevant variations. With a notably high quantum yield of 0.85, and exceptional brightness and photostability, mTFP1 stands as an ideal choice for serving as a FRET donor, particularly in conjunction with acceptor yellow or orange fluorescent proteins (Ai et al., 2006).

***ECFP.*** Enhanced cyan fluorescent protein (ECFP) exhibits pH sensitivity within the pH 5.0 to pH 7.0 range. The fluorescence lifetime of intracellular ECFP undergoes substantial changes (32%) within this pH range, enabling precise pH measurements with an accuracy of less than 0.2 pH units. The use of ECFP fused with chromogranin A (CgA) has been mentioned as a successful application for measuring the pH of secretory granules of cells and analysing pH variations in response to ammonium chloride exposure (Poëa-Guyon et al., 2013).

### Other pH sensors

**FIGURE 1:**
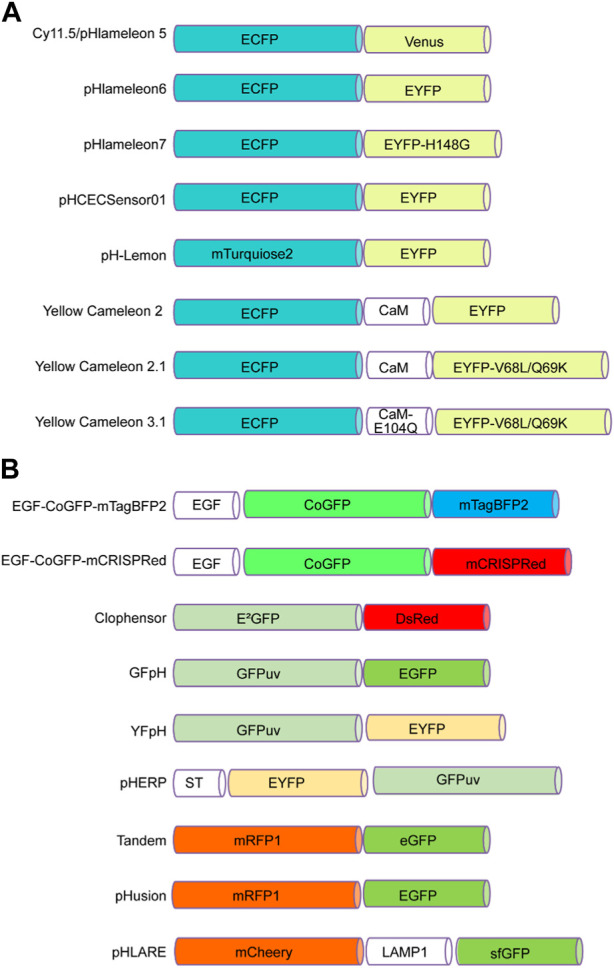
FRET-based pH sensors utilizing pairs of pH-sensitive fluorescent proteins. **(A)** These pairs consist of a cyan fluorescent protein at the N-terminus, such as ECFP or mTurquoise2, and an enhanced yellow fluorescent protein at the C-terminus, such as Venus, EYFP, or EYFP mutants such as EYFP-H148G and EYFP-V68L/Q69K. **(B)** Alternatively, the N-terminus can incorporate green fluorescent proteins such as CoGFP, E^2^GFP, GFPuv, or EYFP, while the C-terminus can contain blue fluorescent proteins such as mTagBFP2, as well as red fluorescent proteins such as RFP1 and mCherry. Green fluorescent proteins such as EGFP, GFPuv, eGFP, and sfGFP are also options.

To date, many genetically encoded fluorescent sensors have been found (Kim et al., 2021). For instance, FRET-based biosensors can sense cAMP, cGMP, glutamate, Ca^2+^, H^+^, and Cl^+^ concentrations (VanEngelenburg and Palmer, 2008). In the following paragraph, we summarize several FRET-based pH biosensors that bind two fluorescent proteins, one of which is sensitive to pH changes (Figure 1).

***pHlameleons.*** pHlameleons represent a type of pH sensor that relies on FRET for precise pH imaging. The evolution of pHlameleons involved substituting N-terminally truncated Venus (Cy11.5/pHlameleon5) with comparably truncated EYFP (pHlameleon6) and EYFP-H148G (pHlameleon7) mutant proteins. This adaptation empowered pHlameleons with heightened sensitivity and an extensive dynamic range, enabling them to detect even subtle pH changes across a wide spectrum of cellular pH values. These sensors capitalize on the FRET phenomenon between acidic and basic fluorescent proteins, which responds to pH shifts. In more acidic cellular environments, structural changes occur in acidic fluorescent proteins, diminishing the FRET effect with basic fluorescent proteins. By detecting and quantitatively analysing this reduced FRET signal using techniques such as fluorescence microscopy, pHlameleons offer valuable insights into fluctuations in intracellular pH levels. Furthermore, pHlameleons can be further refined and tailored to specific research needs through genetic modifications (Esposito et al., 2008).

***pHCECSensor01.*** pHCECSensor01 is an engineered pH sensor composed of a chimeric membrane protein that fuses pH-sensitive EYFP with pH-insensitive ECFP. This unique combination enables ratiometric pH measurements, and with a pKa value of 6.5 ± 0.04, the sensor becomes capable of discerning pH fluctuations within the basolateral spaces of epithelial cells. The effectiveness of the sensor was demonstrated in Madin-Darby canine kidney (MDCK) cells, where it accurately detected pH fluctuations caused by different stimuli. This approach could be a valuable noninvasive method for monitoring extracellular pH changes in various tissues in living organisms (Urra et al., 2008).

***pH-Lemon.*** pH-Lemon stands as a pioneering genetically encoded pH probe that melds the pH-stable cyan fluorescent protein variant mTurquoise2, with the exceptional pH-sensitive enhanced yellow fluorescent protein EYFP (Burgstaller et al., 2019). Under acidic conditions, protonation of the pH-Lemon prompts a significant reduction in yellow fluorescence. Simultaneously, there was an increase in cyan fluorescence attributed to a decrease in FRET efficiency. In addition, the pH-Lemon demonstrated a marked alteration in the pH-dependent fluorescence lifetime. This characteristic positions it for use in fluorescence lifetime imaging microscopy, offering an alternative avenue for investigating pH levels within acidic cellular compartments (Burgstaller et al., 2019).

***Yellow cameleons.*** Cameleons, created by combining green fluorescent protein variants with calmodulin (CaM) and incorporating pH-sensitive EYFP (YC2) and EYFP V68L/Q69K (YC2.1), serve as fluorescent indicators for calcium ions (Ca^2^⁺). These bioengineered constructs are valuable tools for monitoring Ca^2^⁺ levels in diverse biological contexts. Notably, their pH sensitivity is more pronounced at elevated Ca^2^⁺ levels than at zero Ca^2^⁺ (Miyawaki et al., 1999).

***EGF-CoGFP-mTagBFP2/mCRISPRed.*** The two tandem variants exhibited remarkable pH sensitivity within the wide pH range of 4.0–7.5 and can be purified for extracellular applications. Utilized in live-cell microscopy, these pH sensors facilitated the creation of intracellular pH maps, offering insights into pH fluctuations within endocytic vesicles. Moreover, the ability to bind extracellularly to cells harboring EGF receptors allows for pH monitoring within microfluidic chambers. Notably, the dual-emission feature of EGF-CoGFP-mCRISPRed, which holds potential in ratiometric flow cytometry is a valuable method for assessing internalization rates and characterizing protein-drug conjugates in the context of cancer therapy (Karsten et al., 2022).

***ClopHensor.*** The ClopHensor is specifically designed for real-time optical monitoring of chloride ions and pH levels within live cells. ClopHensor exploits the spectral and chemical characteristics of the highly chloride-sensitive GFP variant E^2^GFP anion-binding site linked to DsRed through a flexible 20-amino-acid linker. When ClopHensor is targeted to specific intracellular compartments, it can effectively detect elevated chloride concentrations, particularly within large dense-core exocytosis granules (Arosio et al., 2010). Notably, the H148 and V224L mutations in ClopHensor alter its pKa, increasing alkalinity and enhancing its affinity for chloride ions (Mukhtarov et al., 2013).

***GFpH and YfpH.*** The two pH indicators GFpH (GFPuv-EGFP) and YfpH (GFPuv-EYFP) use two different green fluorescent protein variants and operate in both single-excitation/dual-emission and dual-excitation/single-emission modes through FRET. These indicators were dependent on pH, with pKa values of 6.1 and 6.8. They were successfully used to measure and visualize pH changes in the cytosol and nucleus of cultured cells and near a membrane protein during endocytosis. These probes are valuable tools for monitoring the pH in organelles and around specific proteins, aiding in the analysis of cellular functions (Awaji et al., 2001).

***pHERP.*** The pH-sensitive excitation ratiometric green fluorescent protein (pHERP) was created by fusing EYFP and GFPuv with the peptide linker GGGLEDPRVPVEK. This pHERP was directed to the Golgi using sialyltransferase (ST). The measurement of the Golgi pH (pHG) in HeLa cells expressing ST-pHERP revealed a pHG of 6.4 (Chandy et al., 2001).

***Tandem.*** Tandem is a protein with dual fluorescence properties that comprises a pH-sensitive fluorophore (eGFP; green) and a pH-independent fluorophore (mRFP1; red). This unique composition makes Tandem a versatile pH sensor and delivery indicator within cells. The fusion protein emits both red and green fluorescence, enabling the monitoring of nanoparticles and confirming effective protein delivery. At pH 7.4, both red and green fluorescence are present. Under acidic conditions (pH 4.5–5.0) within lysosomes, green fluorescence diminishes due to eGFP protonation, while red fluorescence remains. The nanoparticles enter cells through endocytosis, move to lysosomes and are observed to colocalize through microscopy. The Tandem fusion protein offers a versatile approach for tracking bioactive nanocarriers, aiding the understanding of their intracellular routes, particularly when delivered by calcium phosphate nanoparticles. This study highlights the necessity of carriers such as nanoparticles for effective cellular uptake of proteins and DNA plasmids (Kollenda et al., 2020).

***pHusion.*** pHusion consists of the tandem concatenation of enhanced green fluorescent protein (EGFP) and monomeric red fluorescent protein (mRFP1) with the short peptide linker AVNAS to obtain a 1:1 stoichiometry, permitting ratiometric measurements of pH changes, where mRFP1 functions as an intramolecular reference (Gjetting et al., 2012).

***pHLARE.*** The pHLARE (pH Lysosomal Activity Reporter) is an innovative biosensor engineered for measuring lysosomal pH (pHlys) in a ratiometric manner. Constructed from rat LAMP1, a transmembrane protein located in lysosomes, features superfolder GFP (sfGFP) attached to its amino-terminus. sfGFP, a GFP variant with a pKa value of approximately 5.9, grants pHLARE the capacity to function within lysosomes across a dynamic pH range spanning 4.0 to 6.5. Its stable expression in cells is achievable. By utilizing pHLARE, researchers successfully observed a significant pHlys reduction upon inhibiting the mammalian target of rapamycin complex 1 (mTORC1). This finding is particularly relevant because that lysosomes are increasingly recognized as promising therapeutic targets (Webb et al., 2021).

## Mechanisms of pH sensing and fluorescence

This section focuses on the detailed mechanisms by which pH-sensitive fluorescent proteins undergo changes in fluorescence intensity or spectral shifts in response to pH variations. We discuss the role of key residues and structural variations within these proteins, such as changes in histidine residues and pH-sensitive chromophores, in mediating pH-dependent fluorescence. The chromophore structural variations in green, yellow, orange, and red FPs have been reported (Shaner et al., 2007), and the chemical diversity of chromophores in fluorescent proteins has also been summarized (Lukyanov, 2022). Additionally, we explored how protein engineering and mutagenesis techniques have facilitated the development of improved pH-sensitive variants with enhanced dynamic ranges and photophysical properties. We delve into the underlying molecular mechanisms that govern their pH sensitivity, shedding light on the intricate conformational changes and protonation/deprotonation events that occur (Bizzarri et al., 2009).

The following is a list of pH-sensitive proteins whose crystal structures have been resolved to date.

### Structural analysis of GFP S65T at low pH and high pH

The X-ray structures of the GFP S65T variant at both low pH 4.6 (PDB: 1C4F) and high pH 8.0 (PDB: 1EMG) revealed structural changes related to the titration of the phenolic hydroxyl group on the chromophore. These changes include the rotation of the Thr203 side chain, the breaking of a hydrogen bond between the chromophore and Thr203, and the loss of a hydrogen bond between His148 and the chromophore at low pH. These findings confirm the protonation of the phenolic end of the chromophore but do not provide evidence for the titration of a second group on the chromophore, such as the imidazolinone ring nitrogen. These results support a model in which the S65T chromophore exists in a pH-dependent equilibrium between neutral and anionic forms, deprotonating with the heterocyclic ring nitrogen. This model may apply to other GFP variants, although exceptions are possible depending on the specific configuration near the chromophore (Elsliger et al., 1999).

### Structural analysis of deGFP1 at low pH and high pH

The X-ray structures of deGFP1 (a green fluorescent protein variant) crystals at both low pH 5.5 (PDB: 1JBY) and high pH 9.0 (PDB: 1JBZ) were performed by molecular replacement and refined at 1.8 and 1.5 Å resolution, respectively. The crystal structures were determined and compared to those of S65T GFP. The barrel-like motif characteristic of GFP was maintained at both pH values. The position of the Cys203 sulfur atom in deGFP1 corresponds to the usual position of the Thr203 carbon atom. The structure does not reveal a proton relay network resembling that found in wild-type GFP. The arrangement of hydrogen bonds around the chromophore is described, and differences in side chain orientations and hydrogen bond configurations at low and high pH values are highlighted. The rearrangement of specific residues in response to pH changes, especially those near position 148, is considered essential for the structural changes observed in deGFP1. These changes are not due to crystallization conditions and appear to be pH-dependent (Hanson et al., 2002).

### Structural analysis of mNeonGreen at low pH and high pH

The study suggested that the X-ray radiation-induced loss of fluorescence in mNeonGreen crystals at pH 4.5 (PDB: 5LTP) and high pH 8.0 (PDB: 5LTR) is due to protonation of the chromophore phenolate group and the separation of resonant electron clouds, resulting in a blueshift of the absorption maximum into the UV region and a loss of absorbance in the visible region. A structural comparison between two fluorescent proteins, lanYFP and mNeonGreen, highlighted the successful evolution process involving rational design and directed evolution. Key mutations disrupting oligomer interfaces were identified, and the authors explained how certain mutations aimed at restoring fluorescence caused rearrangements near the chromophore, leading to a more constrained environment that favors fluorescence. This rearrangement is associated with a small blueshift in the excitation and emission maxima of mNeonGreen compared to lanYFP (Clavel et al., 2016).

### Structural analysis of DendFPs

The green form of DendGFP (λex/λem = 494/506 nm) (PDB: 5EXB) and the photoconverted red form of DendRFP (λex/λem = 560/578 nm) (PDB: 5EXC) of the DendFP structure were solved at 1.81 and 2.14 Å resolution, respectively. The X-ray structure revealed that DendFP undergoes irreversible photoconversion from green to red fluorescence upon UV and blue light irradiation. This transformation involves a structural change, specifically the cleavage of the peptide backbone and the formation of a terminal carboxamide group, which extends the conjugation of the chromophore system and leads to red fluorescence. The paragraph also reports the three-dimensional structures of both the native green and photoconverted red forms of DendFP. Additionally, these findings highlight the role of specific amino acid positions, such as Ser142 and His193, in influencing the photoconversion rate, emphasizing the importance of hydrogen bonding between the chromophore and the Gln116 and Ser105 clusters for this process (Pletneva et al., 2016).

### Structural analysis of Dendra2

The X-ray structure of the green species Dendra2 (PDB:2VZX) is similar to that of related proteins, EosFP and Kaede. The structural changes in Dendra2, which involve mainly Arg66 and the water molecule W4, differ from those in EosFP and Kaede, affecting the charge stabilization on the imidazolinone ring. This structural change leads to a blueshift in the absorption and emission bands and higher pK values for the hydroxyphenyl moiety of the chromophore. The action spectrum of Dendra2 aligns with the neutral species absorption band, explaining why the photoconversion of Dendra2 is greater than that of EosFP, particularly at physiological pH (Adam et al., 2009).

### Structural analysis of mTFP1

The crystal structure of mTFP1 (PDB: 2HQK) was solved at a resolution of 1.19 Å at pH 5.1, confirming its monomeric nature and revealing an unusually distorted chromophore conformation. With a high quantum yield, mTFP1 is suitable for use as a replacement for ECFP or Cerulean in FRET applications with yellow or orange FP acceptors. The structure of mTFP1 revealed that it has the typical ‘β-can’ motif found in Aequorea GFP and its homolog. Structural comparisons with other fluorescent proteins, such as EosFP, dsFP583, and amFP486, revealed high sequence identity and slight structural deviations. The crystal packing of mTFP1 is consistent with its engineered monomeric nature, unlike that of tetrameric Anthozoa FPs. The locations of the mutations in the mTFP1 structure showed that external mutations are involved in disrupting protein-protein interfaces, while internal mutations affect side chain packing and internal hydrogen bond networks (Ai et al., 2006).

**FIGURE 2:**
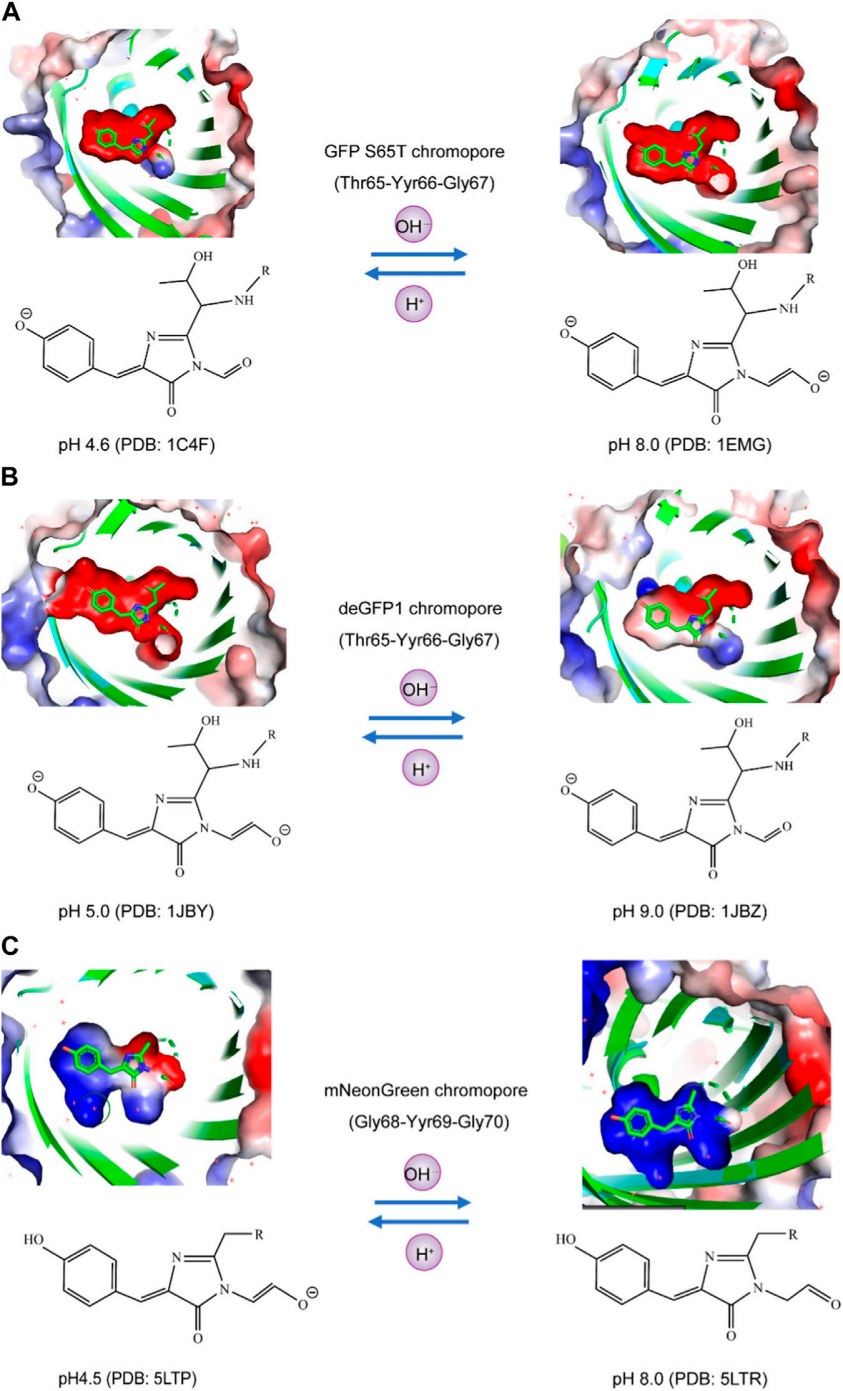
Analysis of the charge states of the phenolic hydroxyl and carbonyl moieties of fluorophores in pH-sensitive fluorescent proteins using the APBS plugin. **(A)** In GFP S65T at both pH 4.6 and pH 8.0, the phenolic groups of the chromophore exhibited a negative charge. Specifically, at pH 4.6, the adjacent carbonyl group, located next to the five-membered ring, remained uncharged, whereas at pH 8.0, it became negatively charged. **(B)** Similarly, deGFP1 displayed similarities with GFP S65T, wherein the phenolic groups of the chromophore carried a negative charge at both pH 5.0 and pH 9.0. However, in contrast to that of GFP S65T, the adjacent carbonyl group, near the five-membered ring, was negatively charged at pH 5.0 but remained uncharged at pH 9.0. **(C)** In the case of mNeonGreen, unlike those of GFP S65T and deGFP1, the phenolic groups of the chromophore did not charge at pH 4.5 or pH 8.0. Nevertheless, akin to deGFP1, the carbonyl group adjacent to the five-membered ring in mNeonGreen had a negative charge at pH 4.5 and remained uncharged at pH 8.0.

Currently, the crystal structures of fluorescent proteins, including GFP S65T, deGFP1, and mNeonGreen, have been elucidated under both high and low pH conditions. The chromophores of these fluorescent proteins exhibit varying charge states of the phenolic group and the carbonyl group adjacent to the five-membered ring at different pH values. For instance, at pH 4.6 and pH 8.0, the phenolic groups of the GFP S65T chromophore are negatively charged. However, at pH 4.6, the carbonyl group adjacent to the five-membered ring is uncharged, while at pH 8.0, it becomes negatively charged (Figure 2A). Similarly, deGFP1 shares similarities with GFP S65T in that at pH 5.0 and pH 9.0, the phenolic groups of the chromophore carry a negative charge. Nevertheless, unlike that in GFP S65T, the carbonyl group adjacent to the five-membered ring in deGFP1 was negatively charged at pH 5.0 and uncharged at pH 9.0 (Figure 2B). In the case of mNeonGreen, at pH 4.5 and pH 8.0, it differs from GFP S65T and deGFP1 in that the phenolic groups of the chromophore do not carry a charge. However, akin to deGFP1, the carbonyl group adjacent to the five-membered ring in mNeonGreen has a negative charge at pH 4.5 and is uncharged at pH 8.0 (Figure 2C). The results of these structural analyses indicate that the chemical properties of these three fluorescent proteins exhibit variations under different pH conditions, particularly in terms of the charge states of the phenolic group and the carbonyl group adjacent to the chromophore. These differences may be associated with the fluorescence properties and pH sensitivity of the biological functions of these fluorescent proteins.

## Applications in organelle pH detection and beyond

This review highlights the diverse applications of pH-sensitive fluorescent proteins in cellular imaging and related fields. Its use in monitoring pH changes within subcellular compartments, and its role in investigating intracellular pH regulation and homeostasis are well known. Additionally, the review explores their applications in studying membrane fusion, protein trafficking, and cellular processes associated with pH fluctuations, as well as their relevance in disease related pathological conditions, such as cancer, neurodegenerative disorders, and infectious diseases.

### Monitoring organelle and subcellular compartment pH

Monitoring organelle pH is a fundamental aspect of cellular research, contributing to a deeper understanding of various cellular processes. A range of methods are employed to achieve this goal, including the use of fluorescent pH-sensitive dyes such as SNARF-1 (Ramshesh and Lemasters, 2018), BCECF nanoparticles, lipobeads and microspheres (Han and Burgess, 2010), microelectrodes (Sommer et al., 2000), and fluorescent proteins (Benčina, 2013). Subcellular localization of monomeric fluorescent protein fusions with targeting proteins can be visualized through widefield fluorescence imaging (Shaner et al., 2007). Genetically encoded pH-sensitive sensors have also been used for monitoring intracellular pH, as discussed in several review papers (Nehrke, 2006; Benčina, 2013; Martynov et al., 2018; Freeman et al., 2023).

Most organelles, except for the nucleus, have their own specific pH associated with the processes that occur in these compartments. The nuclear membrane, which is porous to protons, maintains a pH (pHN) similar to that of the surrounding cytosol. The cytoplasmic and nuclear pH was monitored by several pH-sensitive proteins, the results of which are summarized in Table 2. Cytosolic and nuclear pH levels have been monitored using pH-sensitive proteins, including PrpHluorin (Shen et al., 2013) and pHluorin (Karagiannis and Young, 2001), which have a nuclear location sequence (NLS). Other nuclear markers have been reported for histone H2B (Musinova et al., 2011; Liu Y. et al., 2021), LSD1 (Jin et al., 2014), and fibrillarin (Ochs et al., 1985; Amin et al., 2007; Tiku et al., 2018). The data in Table 2 reveal that the cytosolic pH (pHC) and pHN for resting cells typically range between 7.2 and 7.7. pH measurements using pHGFP and pHusion show pHC values between 6.5 and 7.3.

**TABLE 2:** pH-sensitive FPs targeted to organelle and subcellular compartments.

Organelle	Sensors	pH	References
Cytosol	E^2^GFP	7.2–7.5	Bizzarri et al. (2006), Raimondo et al. (2012)
	deGFP4	7.4–7.7	Raimondo et al. (2012)
	GFP-F64L/S65T	7.4	Kneen et al. (1998)
	EYFP	7.4	Llopis et al. (1998)
	pHluorin	7.0–7.5	Karagiannis and Young (2001), Ullah et al. (2013)
	pHluorin2	7.0–7.5	Ayer et al. (2013), Valkonen et al. (2013)
	PrpHluorin	7.3	Shen et al. (2013)
	SypHer	7.2	Poburko et al. (2011)
	pHRed	7.4	Tantama et al. (2011)
	pHERP	7.2–7.5	Chandy et al. (2001)
	pHGFP	6.5–7.3	Moseyko and Feldman (2001)
	pHusion	6.4	Gjetting et al. (2012)
	Dendra2	7.40 ± 0.05	Pakhomov et al. (2017)
	hCNT3-mNectarine	6.5–7.5	Johnson et al. (2009)
	ClopHensor wild-type/ClopHensor (H148G/V224L)	7.3–7.7	Arosio et al. (2010), Mukhtarov et al. (2013)
Nucleus	NLS-PrpHluorin	7.2	Shen et al. (2013)
	pHluorin-NLS	7.3	Karagiannis and Young (2001)
Mitochondria	mtAlpHi (COX-IV (1–12aa))	8.1	Abad et al. (2004)
	mitoDendra2	7.40 ± 0.05	Pakhomov et al. (2017)
	MitoSypHer (COX-VIII (1–25aa))	7.6	Poburko et al. (2011)
	ECFP-mito (COX-IV (1–12aa))	8.0	Llopis et al. (1998)
	EYFP-mito (COX-IV (1–12aa))	7.9	Llopis et al. (1998)
	mito-pHluorin (COX-IV (1–12 aa)-RSGI linker)	7.7	Vijayvergiya et al. (2004)
	COX8-GFP-F64L/S65T	>7.5	Kneen et al. (1998)
	COX8-pHRed	8.0	Tantama et al. (2011)
	2COX8-pH-Lemon	-	Burgstaller et al. (2019)
	COX4-pHluorin	7.7	Ayer et al. (2013)
	Mito-PpHluorin	8.1	Shen et al. (2013)
Trans-Golgi	GalT-RpHluorin2	5.9 ± 0.07	Linders et al. (2022)
Medial/Trans-Golgi	GT-EGFP/ECFP/EYFP (B4GALT1 (N-terminal 1–81aa))	6.6/6.8	Llopis et al. (1998), Rivinoja et al. (2006)
Medial/Trans-Golgi	GT-EGFP/ECFP/EYFP (B4GALT1)	6.4	Lázaro-Diéguez et al. (2006)
Medial/Trans-Golgi	ST-pHERP (2,6-sialyltransferase (1–70 aa))	6.4–6.6	Chandy et al. (2001)
Medial-Golgi	MGAT2-RpHluorin2	6.1 ± 0.07	Linders et al. (2022)
Cis-Golgi	ManI-PpHluorin	6.8 ± 0.2	Shen et al. (2013)
Trans-Golgi network	TGN38-pHluorin	6.21 ± 0.39	Miesenböck et al. (1998)
	TGN38-pHluorin	6.4	Disbrow et al. (2005)
	PpHluorin-BP80	6.3	Shen et al. (2013)
Endoplasmic Reticulum	pHluorin-KDEL	-	Khiroug et al. (2009)
	GFP-F64L/S65T-SEKDEL	-	Kneen et al. (1998)
	CaR-pH-Lemon-KDEL	-	Burgstaller et al. (2019)
	PpHluorin-HDEL	7.1	Shen et al. (2013)
	ER-RpHluorin2	7.2 ± 0.08	Linders et al. (2022)
Peroxisome	pHluorin-SKL	6.9–7.1/8.0	Jankowski et al. (2001), Ayer et al. (2013)
	PrpHluorin-SRL	8.4	Shen et al. (2013)
Lysosome	LAMP1-RpHluorin2	4.7 ± 0.15	Linders et al. (2022)
	mTFP1	∼4.1	Chin et al. (2021)
Endosome	Cellubrevin-pHluorin	5.51 ± 0.66	Miesenböck et al. (1998)
	TFR-mApple	5.1–6.8	Rennick et al. (2022)

The pH of the resting mitochondrial matrix (pHM) is notably alkaline, ranging from 7.2 to 8.1, as shown in Table 2. Previously, pHluorin, ECFP, EYFP, and ECFP pH sensors, which have a pKa of approximately 7, have been used to measure pHM. These sensors are insensitive to small pH changes at pHM; therefore, pHM dynamics are easily missed. Recent improvements in live cell fluorescence imaging have revealed that proton concentrations rapidly fluctuate within individual mitochondria, as determined using mitoSypHer (Poburko et al., 2011). The pKa of ratiometric mitoSypHer is approximately 8, which is similar to the pKa of pHRed (Tantama et al., 2011), mtAlpHi (Abad et al., 2004), and pHTomato (Li and Tsien, 2012), and they are well suited for pHM measurements. However, specific pH measurements within subcellular mitochondrial compartments, such as cristae and the intermembrane space, have not been performed. To achieve precision, pH-sensitive proteins are combined with mitochondrial markers such as Tom20 (Yano et al., 1998; Bhagawati et al., 2021), VDAC (Shoshan-Barmatz et al., 2010; Camara et al., 2017; Shteinfer-Kuzmine et al., 2022), cytochrome c oxidase subunit 8 (Cox8) (Ross, 2011; Kadenbach and Hüttemann, 2015), Oxa1 (Stiburek et al., 2007; Sato and Mihara, 2009; Sato and Mihara, 2010), HSP60 (Zhou et al., 2018), g subunit (Gu et al., 2019), 6.8 PL (Gu et al., 2019), ferrochelatase (Nagaraj et al., 2009; Watanabe et al., 2013), and mitofilin (Madungwe et al., 2018).

To assess the pH of reticular organelles such as the endoplasmic reticulum, signal peptides such as KEDL and HDEL (Gomord et al., 1997) are commonly used when fused with the pH-sensitive protein pHluorin (Reifenrath and Boles, 2018). The measured pH of the endoplasmic reticulum (pHER) is found to be very close to that of the cytosol (Shen et al., 2013). Markers such as Glimp63 (Zheng et al., 2022), Sec61 (Zheng et al., 2022), Calreticulin (Michalak et al., 2009; Houen et al., 2021), and Calnexin (Ryan et al., 2016), can be employed for localization and pH testing. However, tagging subcellular domains of the Golgi apparatus can be challenging. Many Golgi proteins briefly reside in the endoplasmic reticulum, indicating cycling between the Golgi and endoplasmic reticulum (Storrie, 2005). The pH of the cis-Golgi is 6.8 ± 0.2, which is slightly more acidic than that of the endoplasmic reticulum (7.1 ± 0.4) (Shen et al., 2013). The trans/medial-Golgi pH ranges from 6.4 to 6.81, and the pH can be measured using β-1,4-galactosyltransferase (GT) fused with pH-sensitive EYFP (Llopis et al., 1998; Lázaro-Diéguez et al., 2006; Rivinoja et al., 2006). Various markers, including B4GALT1/ST6GAL1 (Qasba et al., 2008; Hassinen et al., 2010; Peanne et al., 2011; Khoder-Agha et al., 2019), and GalT (Linders et al., 2022), as well as TGN38 (Bos et al., 1993), are employed for trans-Golgi measurements, while the cis-Golgi markers GM130 (Tie et al., 2016; Zheng et al., 2022), GMAP-210 (Infante et al., 1999), GP73 (Bachert et al., 2007; Hu et al., 2011), MGAT2/GnTII (Hassinen et al., 2010), and Giantin (Linstedt and Hauri, 1993; Satoh et al., 2019) are utilized for cis/medial-Golgi measurements.

Lysosomes are acidic organelles, and their pH can be measured using fluorescent proteins sensitive to acidic conditions, such as mTFP1 (FIRE-pHLy) (Chin et al., 2021) and LAMP1 conjugated with RpHluorin (Linders et al., 2022). Additionally, markers such as Dnase2B (Shpak et al., 2008; Ohkouchi et al., 2013), and Cathepsin D (Chai et al., 2019) are used for lysosome localization. Peroxisomes are alkaline organelles, with a pH close to 8.0. The pH of the cells was measured via the fusion of SKL or SRL with pHluorin (Miura et al., 1992; Wolins and Donaldson, 1997; Elleuche and Pöggeler, 2008). In addition to the above signaling peptides, other peroxisome markers, such as Catalase have been identified (Zheng et al., 2022). Endosomes, comprising early and late endosomes, are characterized by markers such as the Cellubrevin (Miesenböck et al., 1998), TFR (Rennick et al., 2022), and EEA1 (Zheng et al., 2022). The pH was determined to be 5.51 ± 0.66 for Cellubrevin-pHluorin and ranged from 5.1 to 6.8 for TFR-mApple, respectively. Additionally, a genetically encoded pH probe called pH-Lemon was designed for live imaging of distinct pH conditions within acidic cellular compartments, particularly lysosomes and endosomes. The pH-Lemon was created by fusing a pH-stable cyan fluorescent protein (mTurquoise2) with the highly pH-sensitive EYFP (Burgstaller et al., 2019). This probe offers reversible and ratiometric responses, making it suitable for monitoring pH dynamics. It also exhibits pH-dependent changes in fluorescence lifetime, enabling its use in fluorescence lifetime imaging microscopy. Fusion of the pH-Lemon to specific cellular markers allows for the visualization of pH variations in different subcellular structures and compartments, providing a valuable tool for studying local pH dynamics in living cells.

### Visualizing synaptic transmission: exocytosis, endocytosis, docking, fusion

Fluorescent proteins exhibiting pH-sensitive fluorescence play a crucial role in visualizing exocytosis and endocytosis processes. Among these fluorescent proteins, the Aequorea green fluorescent protein mutant known as superecliptic pHluorin is highly suitable for such applications. Ratiometric pHluorin is coupled with the vesicle membrane protein VAMP2, which can sort both secretory and synaptic vesicles, facilitating the precise monitoring of transmission at individual synaptic boutons (Miesenböck et al., 1998).

The use of synapto-pHluorin, a variant of pHluorin that is targeted to the synaptic vesicle lumen, has enabled the measurement of dynamic pH changes within vesicles resulting from exocytosis and endocytosis during presynaptic activity. The observed fluorescence changes during action potentials reflect a delicate balance between the brightly fluorescent externalized pHluorin and the darkened, endocytosed, and reacidified vesicles (Sankaranarayanan et al., 2000).

Additionally, a construct called hPTH1R-pHluorin2, in which pHluorin2 is located in the ligand-binding domain of the parathyroid hormone 1 receptor, exhibited a substantial increase in fluorescence upon ligand-induced endocytosis into intracellular vesicles, making it readily detectable by confocal microscopy (Mahon, 2011).

Notably, the pH-sensitive protein superecliptic pHluorin, in conjunction with pH-sensitive organic fluorophores such as carbofluorescein (CFI) and virg1inia orange (VO), which integrate the SNAP-tag into an intraluminal loop of the vesicular acetylcholine transporter VAChT, has played a pivotal role in the detection of single exocytosis events within PC12 cells. Similarly, the ability to detect synaptic vesicle exocytosis and recycling in hippocampal neurons was made feasible through the use of pH-sensitive proteins and the organic fluorophores SEP, CFI, and VO conjugated with VAMP2. These groundbreaking constructs offer a powerful means to visualize both exocytosis and endocytosis, thereby introducing exceptionally bright and innovative sensors for pivotal synaptic transmission processes (Martineau et al., 2017).

Moreover, the incorporation of pHuji, a red fluorescent protein renowned for its pH sensitivity, in conjunction with SEP enables the simultaneous use of two-color imaging. This combination of methods helps individuals detect exocytosis and endocytosis events associated with clathrin-mediated internalization, involving proteins such as the transferrin receptor (TfR) and the β2 adrenergic receptor (β2AR). In brief, the ability to detect exocytosis and endocytosis events allows for the pairing of TfR-SEP with TfR-pHuji, which can also be paired with other pH-sensitive red fluorescent proteins, such as TfR-pHTomato and TfR-pHoran4. Furthermore, this approach facilitates the observation of differential sorting during clathrin-coated vesicle (CCV) formation through the utilization of TfR-pHuji and SEP-β2AR (Shen et al., 2014).

An additional noteworthy creation is SypHTomato, which was generated by merging pHTomato with synaptophysin. This innovative construct effectively reports activity-driven exocytosis akin to that of green reporters. When coupled with the GFP-based indicator GcaMP3, SypHTomato permits the simultaneous monitoring of transmitter release and presynaptic Ca^2+^ fluctuations within individual nerve terminals (Li and Tsien, 2012).

Considering that prior research has indicated the widespread use of SEP for single-vesicle exocytosis imaging, it is essential to acknowledge its limitations in visualizing the docking step. This is primarily due to the low fluorescence signal within vesicles, which renders them unsuitable for monitoring the docking process. In stark contrast, pHmScarlet, among various other red fluorescent proteins, exhibited increasing pH sensitivity. Furthermore, compared with that of SEP, the redshifted emission wavelength of SEP maintains ample spatial resolution for elucidating the ring structure of vesicle fusion pores. This capacity is harnessed through the use of Hessian structured illumination microscopy (Hessian-SIM), which enables the simultaneous tracking of both vesicle docking and fusion events. The amalgamation of pHmScarlet with SEP introduces the potential for dual-color imaging, allowing for the observation of two distinct exocytosis and fusion events (Liu A. et al., 2021).

### Understanding drug effects and disease progression

pH-dependent fluorescent lifetime imaging microscopy (pHLIM) combined with deep learning was used to accurately measure the subcellular pH of individual vesicles. A pH-sensitive protein called mApple was used to target specific cellular compartments such as the cytosolic mApple, endosomes (TfR-mApple), and lysosomes (TMEM106b-mApple). This approach allows for the rapid and accurate detection of pH changes induced by drugs, such as bafilomycin A1 and chloroquine, and provides a valuable tool for understanding drug effects and disease progression, particularly in the context of cargo release from therapeutic delivery systems. The acidifying endo/lysosomal pathway represents a promising avenue for improving the precision of drug delivery (Cheng et al., 2013). Utilizing pH-responsive systems, including nanomaterials (Deirram et al., 2019) and linkers such as acetal (Gillies et al., 2004; Cohen et al., 2011), can exploit the acidic environment to enhance cargo delivery to specific pH compartments in the cellular trafficking pathway. Consequently, visualizing the native pH of subcellular locations where these materials traffic is crucial for optimizing the design of these systems. This approach overcomes the limitations associated with traditional pH measurement methods and fluorophores, making it a simple and quantitative approach for studying subcellular pH dynamics (Rennick et al., 2022).

### Detecting cell death and apoptosis processes

Several researchers have developed a novel cell death assay based on changes in cytosolic pH as an indicator of programmed cell death (PCD) in plants. The authors used the extinction of YFP fluorescence at low pH to monitor cytosolic acidification during PCD, and the fluorescence was recovered when the pH was restored to neutral, indicating no YFP degradation. This nondestructive assay allows time-lapse studies and visualization of subcellular localization changes during PCD. The authors suggest that this tool can aid in the study of the genetic regulation and cell biology of PCD in plants, cautioning other researchers to consider the impact of pH changes on fluorescent reporters when conducting similar studies (Young et al., 2010).

The use of pH-sensitive green fluorescent protein (GFP) and YFP(H148G) (Wachter et al., 1998) (hereafter referred to as pH-GFP) enables the observation of fluctuations in intramitochondrial and cytosolic pH levels in response to mitochondria-dependent apoptotic triggers, such as Bax, staurosporine, and ultraviolet irradiation. Mitochondria play a crucial role in triggering apoptosis by releasing caspase activators, including cytochrome c (cytC). These authors found that mitochondria-dependent apoptotic stimuli lead to rapid changes in the intracellular pH, mitochondrial alkalinization and cytosolic acidification, followed by cytC release, caspase activation, and mitochondrial swelling and depolarization. These events are specific to mitochondria-dependent stimuli and are not induced by mitochondria-independent apoptotic triggers. The authors found that the acidification of the cytosol induced by mitochondria may be a key factor in caspase activation during apoptosis, and this effect was supported by experiments with protonophores and yeast cells (Matsuyama et al., 2000).

### Monitoring nucleoside transport

The pH-sensitive fluorescent protein mNectarine was used to monitor H^+^/uridine cotransport in cultured mammalian cells, particularly focusing on the human concentrative nucleoside transporter hCNT3. By fusing mNectarine to hCNT3, they were able to measure the changes in pH at the intracellular surface of hCNT3. The results indicate that H^+^/uridine cotransport occurs under acidic conditions, providing direct evidence of this process. Additionally, the study suggested that under acidic, Na^+^-containing conditions, both Na^+^ and H^+^ ions are transported along with uridine, while under Na^+^-free acidic conditions, only H^+^ ions are transported with uridine. This approach of using the mNectarine fusion to monitor nucleoside transport is considered simple and effective for studying the activity of H^+^-coupled transport proteins (Johnson et al., 2009).

**FIGURE 3:**
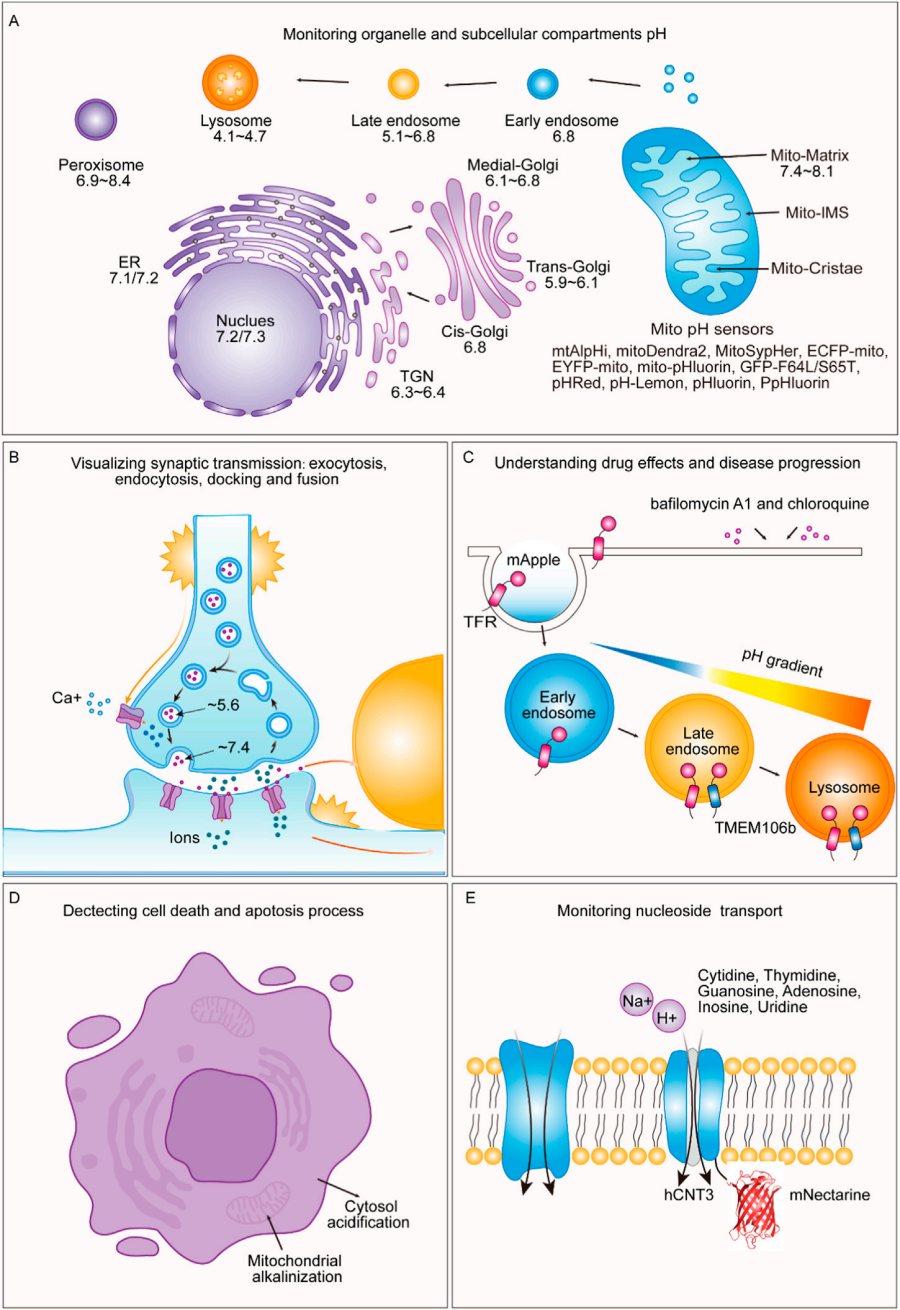
Applications of pH-sensitive proteins in organelle pH detection and beyond. **(A)** Our review shows the pH levels within distinct subcellular compartments in typical cells. We exclusively report pH values obtained using genetically encoded pH-sensitive fluorescent proteins as indicators. These values have been collated from various references and are cross-referenced in Table 2. **(B)** During exocytosis, mature vesicles fuse with the plasma membrane, causing a rapid shift in internal pH from an acidic environment (approximately pH 5.6) to a slightly alkaline extracellular environment (approximately pH 7.4). Notably, pH-sensitive proteins such as VAMP2-ratiometric pHluorin, synapto-pHluorin, hPTH1R-pHluorin2, TfR-pHuji, SEP-β2AR, and SypHTomato enable the visualization of exocytosis, endocytosis, docking, and fusion events in synaptic transmission and secretion. **(C)** pH-sensitive fluorescent proteins are valuable for understanding drug effects and disease progression. By utilizing a pH-sensitive protein called mApple, specific cellular compartments, such as the cytosolic mApple, endosomes (TfR-mApple), and lysosomes (TMEM106b-mApple) can be targeted. This approach allows for the rapid and precise detection of pH changes induced by drugs such as bafilomycin A1 and chloroquine. **(D)** pH-sensitive fluorescent proteins can detect processes related to cell death and apoptosis triggered by mitochondria-dependent apoptotic stimuli, leading to rapid changes in the intracellular pH, including mitochondrial alkalinization and cytosolic acidification. **(E)** pH-sensitive fluorescent proteins can be employed to monitor nucleotide transport. By fusing mNectarine to hCNT3, the changes in pH at the intracellular surface of hCNT3, which is responsible for transporting molecules such as cytidine, thymidine, guanosine, adenosine, inosine, and uridine, can be measured.

Finally, we summarized the diverse applications of pH-sensitive fluorescent proteins, as shown in Figure 3.

### Other applications

deGFP4, E^2^GFP, and the Cl-sensor can also be employed to investigate activity-dependent pH fluctuations at the single-neuron level (Raimondo et al., 2012). pH-sensitive genetically encoded probes such as pHluorin2 have enabled the monitoring of pH within different compartments of *Candida albicans* (Tournu et al., 2017). Additionally, the pH within the cellular cortex of *Drosophila* S2 cells was examined by employing a pHMA sensor-pHluorin fusion linked to the protein moesin, as previously described (Fishilevich et al., 2010).

## Recent advances and future directions

The field of pH-sensitive fluorescent proteins has experienced a surge of innovation in recent years, opening up exciting new avenues for research and expanding their utility. In this section, we highlight some of the recent advances in the field and provide insights into promising future directions.

### Enhanced photostability and specificity

Recent research efforts have been directed toward improving the photostability and pH specificity of fluorescent proteins. These advancements are crucial for extending the usability of these proteins for long-term imaging and high-resolution studies. The development of pH-sensitive variants with reduced photobleaching has the potential to revolutionize long term experiments, especially those requiring super-resolution microscopy.

### Subcellular targeting capabilities

Genetically encoded pH sensors have become more sophisticated in terms of subcellular targeting. The ability to precisely direct these sensors to specific organelles and subcellular compartments is a valuable tool for dissecting complex cellular processes. The future is likely to see the emergence of even more precise targeting strategies, allowing researchers to examine pH dynamics at unprecedented levels of detail.

### Multiplexed imaging and combinatorial approaches

To gain a more comprehensive understanding of cellular processes, there is a growing trend toward multiplexed imaging. Researchers are combining pH-sensitive fluorescent proteins with other fluorescent markers, such as calcium indicators or protein sensors, to investigate complex interactions. This approach is likely to become increasingly common and provide deeper insights into the interplay of pH with other cellular parameters.

### Three-dimensional imaging and super-resolution microscopy

As imaging techniques continue to evolve, the application of pH-sensitive fluorescent proteins in three-dimensional imaging and super-resolution microscopy is expanding. These proteins are now used to study pH dynamics within intricate cellular structures and at nanoscale resolutions. Future developments in this area will allow researchers to uncover previously inaccessible details of pH regulation.

### Applications in optogenetics

The potential of pH-sensitive fluorescent proteins in the emerging field of optogenetics is an area of great promise. By combining pH sensors with light-activated proteins, researchers can precisely manipulate cellular processes triggered by pH changes. This approach holds the potential to revolutionize our ability to modulate biological responses with high spatiotemporal precision.

### Drug discovery and therapeutic interventions

Understanding the role of pH in cellular processes is crucial for drug discovery. pH-sensitive fluorescent proteins can be employed to screen potential drug candidates for their effects on pH-dependent mechanisms. This application has the potential to accelerate the development of therapies for a wide range of diseases.

As the field of pH-sensitive fluorescent proteins continues to advance, the potential for breakthroughs in both fundamental biology and therapeutic interventions becomes increasingly evident. These remarkable tools have paved the way for a deeper understanding of the dynamic pH changes that underlie complex biological processes. With ongoing research and technological advancements, pH-sensitive fluorescent proteins will remain at the forefront of cellular biology, enabling researchers to explore new frontiers and uncover the intricacies of cellular physiology and disease mechanisms.

## Conclusion and outlook

The remarkable versatility of pH-sensitive fluorescent proteins has undoubtedly transformed the landscape of cellular biology and physiology. This review has elucidated the indispensable role of these fluorescent proteins in elucidating the dynamic pH changes that underlie complex biological processes. Progress through the diverse types, mechanisms, and applications of these fluorescent proteins has shown their monumental impact on our ability to understand, visualize, and monitor pH dynamics in living systems.

As we stand on the precipice of further scientific discovery, it is essential to acknowledge the pivotal contributions made by these fluorescent proteins in various domains of cellular research. The ability of these methods to provide real-time, noninvasive measurements of pH alterations within subcellular compartments has been pivotal in deciphering the intricacies of cellular physiology. Insights gained from the study of exocytosis, endocytosis, organelle pH, and subcellular pH localization have significantly advanced our understanding of cellular processes.

Moreover, recent advancements in the field of pH-sensitive fluorescent proteins have opened new horizons. The development of genetically encoded pH sensors with enhanced photostability, specificity, and subcellular targeting capabilities augments their utility. Techniques such as multiplexed imaging, three-dimensional visualization, and super-resolution microscopy are expanding their applications to uncharted territories.

The potential applications of pH-sensitive fluorescent proteins in fields such as drug discovery offer exciting prospects for future research. Exploring the acidifying endo/lysosomal pathway has emerged as a promising avenue for enhancing the precision of drug delivery. Visualizing the native pH of these subcellular locations is crucial for harnessing proteins’ ability to modulate cellular processes precisely through targeted drug delivery. The promise lies in understanding how these proteins, by influencing pH-dependent mechanisms, can lead to breakthroughs in both fundamental biology and therapeutic interventions. However, there are certain limitations associated with pH-sensitive fluorescent proteins. Some exhibit a lower quantum yield, consequently reducing their fluorescence signal intensity. Others exhibit narrow pH response ranges, constraining their applicability for measuring diverse pH environments. Additionally, certain proteins feature a single excitation and single emission characteristic, which may not be ideal for precisely measuring pH levels in various organelles, especially when utilizing radiometric methods.

In conclusion, pH-sensitive fluorescent proteins remain at the forefront of biological research, empowering scientists to unravel the intricacies of cellular physiology and disease mechanisms. With continuous advancements and refinements, these proteins will continue to be indispensable instruments, shedding light on the dynamic changes in pH within living cells and tissues. The future of cellular biology, guided by pH-sensitive fluorescent proteins, holds the promise of uncovering even more profound insights into the dynamic world of pH in biological systems.
